# Effects of pepsin and pepstatin on reflux tonsil hypertrophy *in vitro*

**DOI:** 10.1371/journal.pone.0207090

**Published:** 2018-11-08

**Authors:** Jin Hyun Kim, Si Jung Jang, Jeong Won Yun, Myeong Hee Jung, Seung Hoon Woo

**Affiliations:** 1 Biomedical Research Institute, Gyeongsang National University Hospital, Jinju, Korea; 2 Institute of Health Science, Gyeongsang National University, Jinju, Korea; 3 Department of Otorhinolaryngology-Head and Neck Surgery, Gyeongsang National University Hospital, Jinju, Korea; 4 Beckman Laser Institute, University of California, Irvine, California, United States of America; Universitatsklinikum Freiburg, GERMANY

## Abstract

There is evidence that pepsin can aggravate tonsil hypertrophy. Pepstatin is a potent inhibitor of pepsin activity and could protect patients against reflux tonsil hypertrophy by inhibiting pepsin. We examined the effects of pepstatin on the development of tonsil hypertrophy to investigate pepsin’s role in the pathogenesis of tonsil lesions. We investigated whether pepstatin suppresses pepsin-mediated lymphocyte proliferation in tonsil hypertrophy. Forty-nine children with tonsil hypertrophy and twenty-two adults with tonsillitis were recruited to the study prior to surgery. Tonsil tissue from each patient was harvested and assessed for changes in the number of lymphocytes and macrophages in the presence of pepsin and pepstatin. We found that the proportions of CD4- and CD14-positive cells were significantly lower (*p* < 0.05), but that the proportions of CD19- and CD68-positive cells were significantly higher (*p* < 0.05), in children than in adults. There were significantly more CD4-positive cells after pepsin treatment, but these numbers were reduced by pepstatin. The levels of both interleukin-2 (IL-2) and interferon gamma (IFN-γ) increased significantly in response to pepsin, but were reduced when pepsin was inhibited by pepstatin. The level of IL-10 is reduced in pepsin-treated CD4 cells and the level is restored by pepstatin. IL-2 blocking reduced the increased CD4 cell number by pepsin. But, an additive or a synergic effect is not founded in combined with IL-2 blocking and pepstatin. Pepsin-positive cells did not co-localize with CD20 and CD45 cells, but they were found surrounding CD20- and CD45-positive hypertrophic tonsil cells. Pepsin-positive cells co-localized with CD68-positive cells. It is probable that pepsin from extraesophageal reflux aggravates tonsil hypertrophy and pepstatin exerts a protective effect by inhibiting pepsin activity.

## Introduction

Tonsil hyperplasia is one of the most common indications for tonsillectomy.[1–3] An increase in the total number of lymphocytes also increases tissue volume and enlarges the tonsils.[4, 5] Although many studies have suggested a role for bacteria in tonsil hypertrophy pathogenesis.[6–8], the number of tonsil lymphocytes can increase in the absence of a clinical infection. This suggests that the specific antigens might exist to cause tonsil hypertrophy. [5, 9]

The reflux of gastric contents, termed extraesophageal reflux, can produce a variety of symptoms and aggravate inflammatory disorders, and it is as common in children and infants as it is in adults.[10] An estimated 10% of patients visiting clinics have a reflux-associated disease, and up to 55% of patients with hoarseness have reflux in the laryngopharynx.[11] Extraesophageal reflux is one of the most common factors associated with inflammation of the upper airways. Pepsin, an acid-activated protease that is secreted by stomach chief cells, is an important component of gastric refluxate.[10] There is evidence that pepsin is involved in the pathogenesis of tonsil hypetrophy.[9]

Pepstatin is a potent inhibitor of pepsin activity. Studies with pepstatin the highly specific aspartic-protease inhibitor have been carried out on individual active and proenzymes to assess any enzymic similarities.[12–14] The preventative action of pepstatin upon gastric ulceration in the pylorus-ligated rat has been observed and confirmed protection from pepsin induced ulceration in a rat model.[15, 16]

We already demonstrated that reflux pepsin is one of target in pediatric tonsillar hypertrophy.[17] This study is designed to investigate another therapeutic tool except surgery for treating the pediatric patients with tonsillar hypertrophy and is especially focused to inhibit the effect of pepsin. Therefore, we investigated whether pepsin is involved in lymphocyte proliferation and the inhibitory effects of pepstatin on the development of reflux tonsil hypertrophy.

## Results

### Patients characteristics

All patients underwent physical examination to confirm the diagnosis of tonsillar hypertrophy or chronic tonsillitis. The mean size of tonsil was grade 2.5 in tonsil hypertrophy group and grade 1.0 in chronic tonsillitis group. We obtained the tonsil tissues from 49 children prior to their surgical treatment (29 boys and 20 girls; age range 4–16 years, mean age 8 years) and from 22 adults (9 males and 13 females; age range 17 and over, mean age 31 years) (Table 1).

**Table 1:** The characteristics of patients.

	Children (N = 49)	Adult (N = 22)
Age (years)	8.24±2.03	31.33±3.27
Gender (male: female)	29:20	9:13
Tonsil Size (Grade)	2.5	1.0

### Cell types and numbers in the tonsils of children with hypertrophy

**Fig 1:**
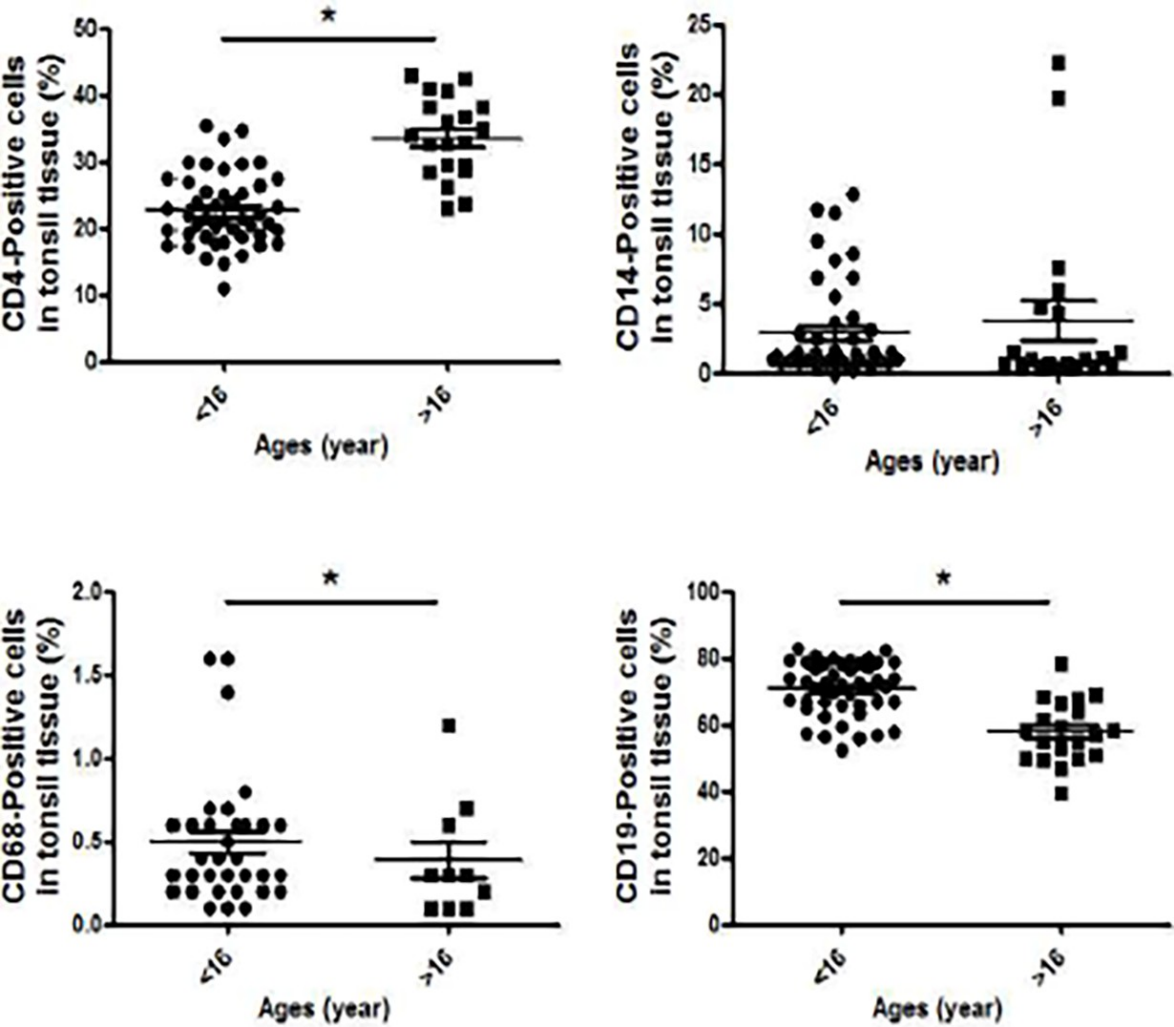
Immune cell population from hypertrophic tonsils and patients with tonsillitis. Tissues were homogenized and washed with cold phosphate-buffered saline, and tonsil cells were stained with anti-CD4, CD14, CD68, and CD19 antibodies to identify T lymphocytes, monocytes, macrophages, and B cells, respectively. Values represent mean ± standard error of the mean (SEM). *p < 0.05 (patients <16 years, n = 46; patients >16 years, n = 20).

On the day of surgery, CD4, CD14, CD68, and CD19-positive cells were identified from tonsil tissue using flow cytometry analysis. As shown in Fig 1, the proportions of CD4-positive cells were significantly lower in children than in adults (children vs. adults, 25.9% vs. 38%, 22.9% vs. 34.2%, *p < 0.05, respectively) but the proportions of CD68- and CD19-positive cells were significantly higher in children than in adults (children vs. adults, 0.49% vs. 0.39%, 70.4% vs. 57.5%, *p < 0.05, respectively). The proportions of CD14-positive cells did not differ between the two groups (children vs. adult, 3.3% vs. 4.7%, *p* > 0.05.). Interestingly, there was a difference between tonsil tissue and the peripheral blood of hypertrophic tonsils. The pediatric peripheral blood samples revealed lymphocytes, CD14-, and CD206-positive cells, there were significantly more lymphocytes than in the adult samples (S1 Fig). These results suggest that lymphocytes might play an important role in tonsil hypertrophy and tonsillitis.

### Tonsil CD4-positive cells respond to pepsin

**Fig 2:**
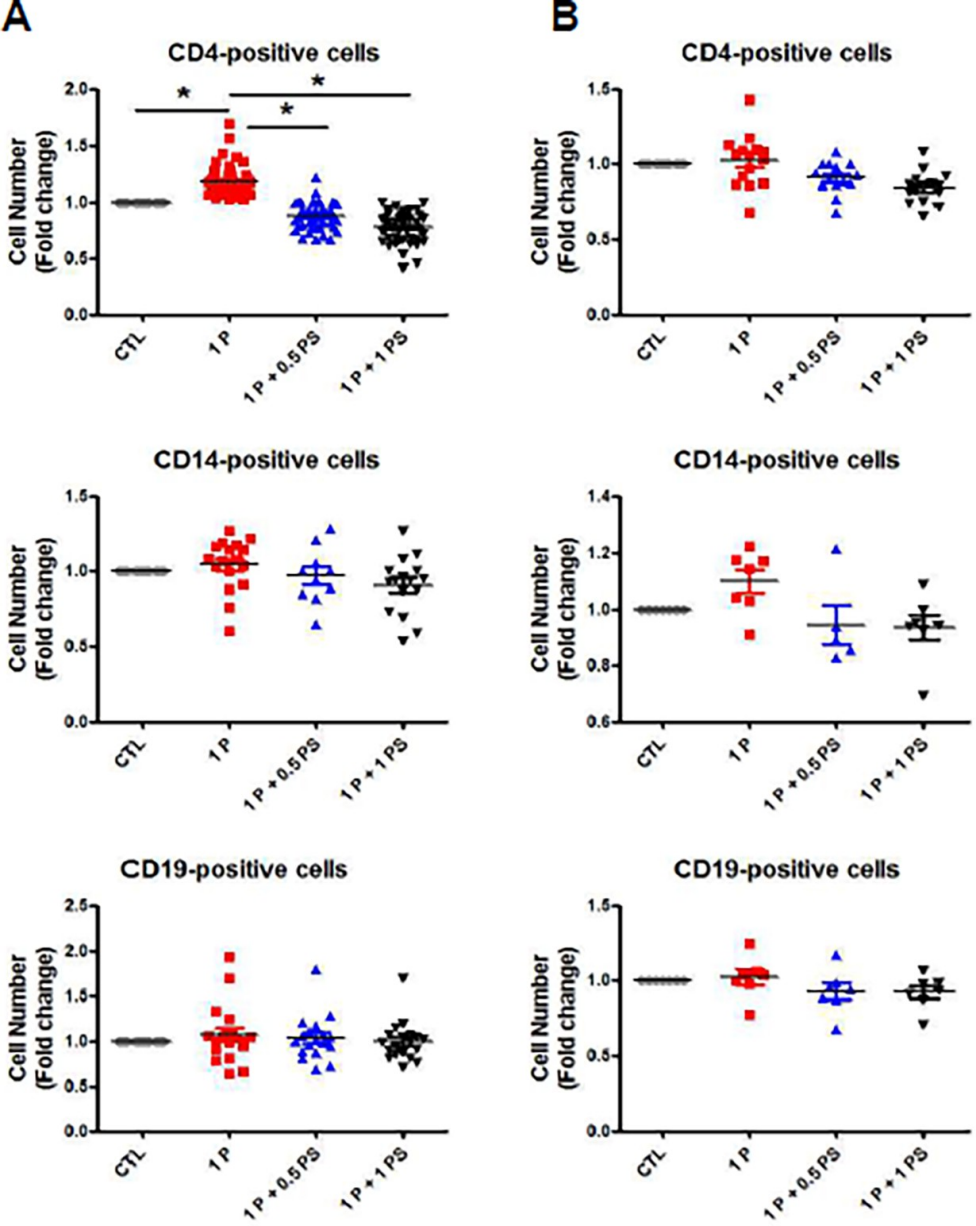
Effect of pepsin on proliferation of cells from patients with tonsil hypertrophy and tonsillitis. Tonsil cells from pediatric patients with hypertrophic tonsils (<16 years. A) and from patients with tonsillitis (>16 years. B) were isolated using magnetic-activated cell sorting (MACS), treated with pepsin and pepstatin for 7 d, and counted. Fold changes were calculated relative to final values in the absence of pepsin and pepstatin (set as “1”). Values represent mean ± SEM. *p < 0.05 (n = 35). 1P, 1.0 μg/ml pepsin; 0.5 PS, 0.5 μg/ml pepstatin; 1 PS, 1.0 μg/ml pepstatin; CTL, no pepsin or pepstatin.

Our previous work demonstrated that extraesophageal pepsin from stomach refluxate can promote tonsil hypertrophy.[9] To identify the cell population responds to pepsin in hypertrophic tonsils, CD4-, CD14-, and CD19-positive cells were purified by magnetic-activated cell sorting (MACS) and cultivated in the presence of pepsin or of pepstatin, which inhibits pepsin (S2 Fig). Purification for tonsillar macrophage was intentionally excluded due to low level of CD68 in the tissue as shown Fig 1A. The number of CD4-positive cells significantly increased when these were treated with pepsin, compared with untreated CD4-positive control cells, but decreased in response to the addition of pepstatin (1 P [pepsin] + 0.5 PS [pepstatin] and 1 P + 1 PS; Fig 2A) in children tonsil tissue. However, there were no statistically significant differences in the numbers of CD14- and CD19-positive cells (Fig 2A). In addition, there were no statistically significant differences among any of the tonsil cell populations in adult tonsil tissue (Fig 2B). These results suggest that CD4 cells respond to pepsin and this response is inhibited by pepstatin. It may be that pepsin is involved in CD4 cell proliferation, and children are more susceptible to pepsin exposure than adults are (S1 Dataset and S2 Dataset).

### Pepsin-stimulated CD4 cell proliferation is mediated by interleukin-2 (IL-2)

**Fig 3:**
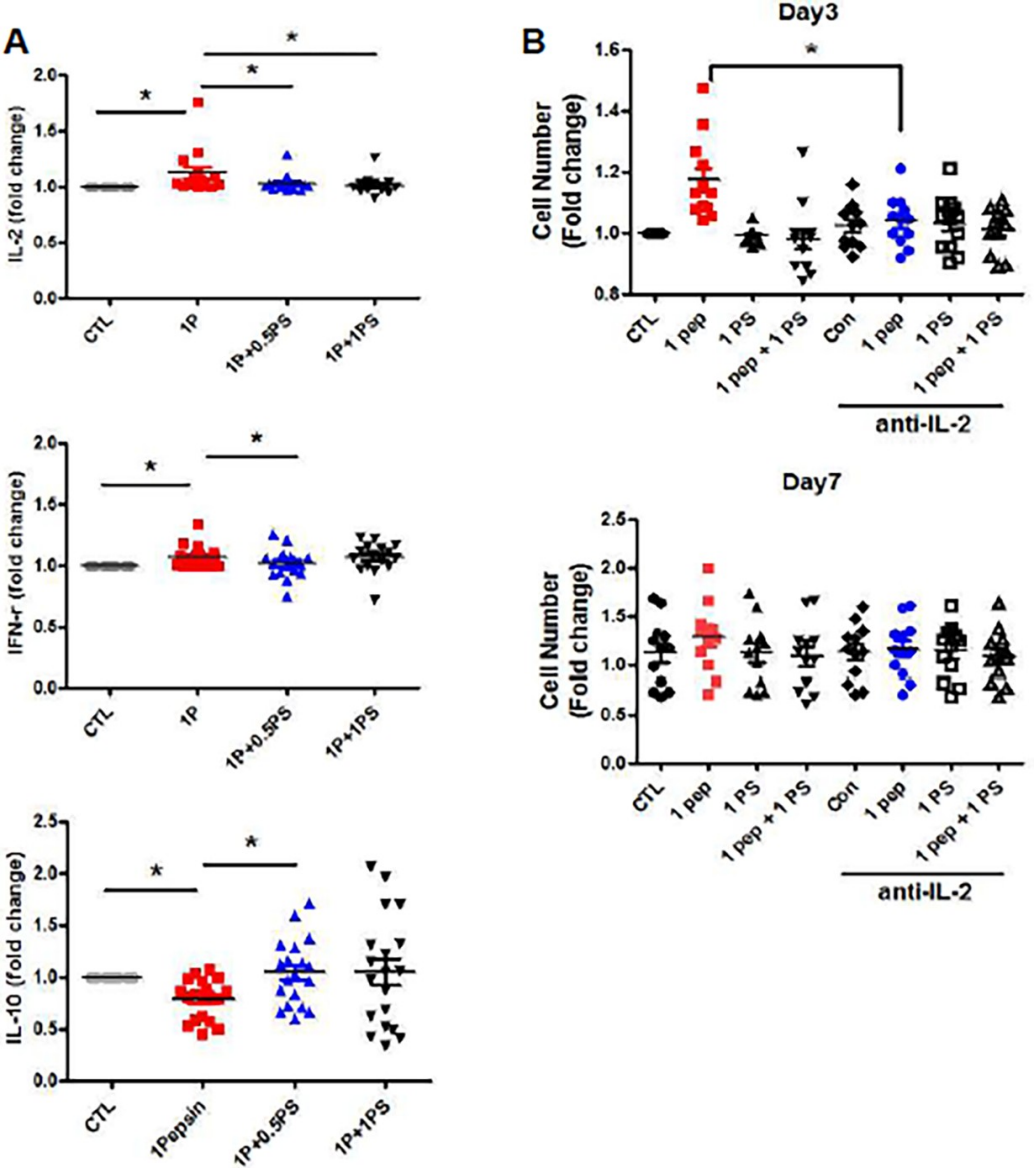
Effect of pepsin on secretion of interleukin-2 (IL-2) and interferon gamma (IFN-γ), and interleukin 10 (IL-10) by CD4-positive cells and anti-pepsin effect of IL-2 blocking. CD4-positive cells were isolated using MACS and treated with pepsin and pepstatin for 7 d. The IL-2, IFN-γ, and IL-10 levels in the culture medium were measured by enzyme-linked immunosorbent assay (ELISA) (A). IL-2 is blocked by IL-2 blocking antibody (anti-IL-2) on day 3 and day 7. The cell number is counted in each treated groups (B). Values represent mean ± SEM. *p < 0.05 (n = 17). 1P, 1.0 μg/ml pepsin; 0.5 PS, 0.5 μg/ml pepstatin; 1 PS, 1.0 μg/ml pepstatin; CTL, no pepsin or pepstatin.

To identify a possible mechanism for pepsin-mediated CD4 cell proliferation, the levels of IL-2 and IFN-γ, cytokines that can stimulate lymphocyte proliferation, were measured in cultivation media by enzyme-linked immunosorbent assay (ELISA). Both IL-2 and IFN-γ levels significantly increased in response to pepsin treatment, but decreased when pepsin was inhibited by pepstatin. The level of IL-10, a representative anti-inflammatory cytokine, is reduced in pepsin-treated CD4 cells and the level is restored by pepstatin. (Fig 3A). IL-2 may be more sensitive to pepsin than IFN-γ and L-10 are. To confirm if pepsin is involved in IL-2-mediated CD4 cell proliferation, IL-2 is blocked with IL-2 blocking antibody (anti-IL-2) (Fig 3B). IL-2 blocking (blue) reduced the increased CD4 cell number by pepsin (red) on day 3. But, an additive or a synergic effect is not found in combined with IL-2 blocking and pepstatin on day 3. There is no positive finding on day 7.

### Expression of pepsin and lymphocytes in hypertrophic tonsils

**Fig 4:**
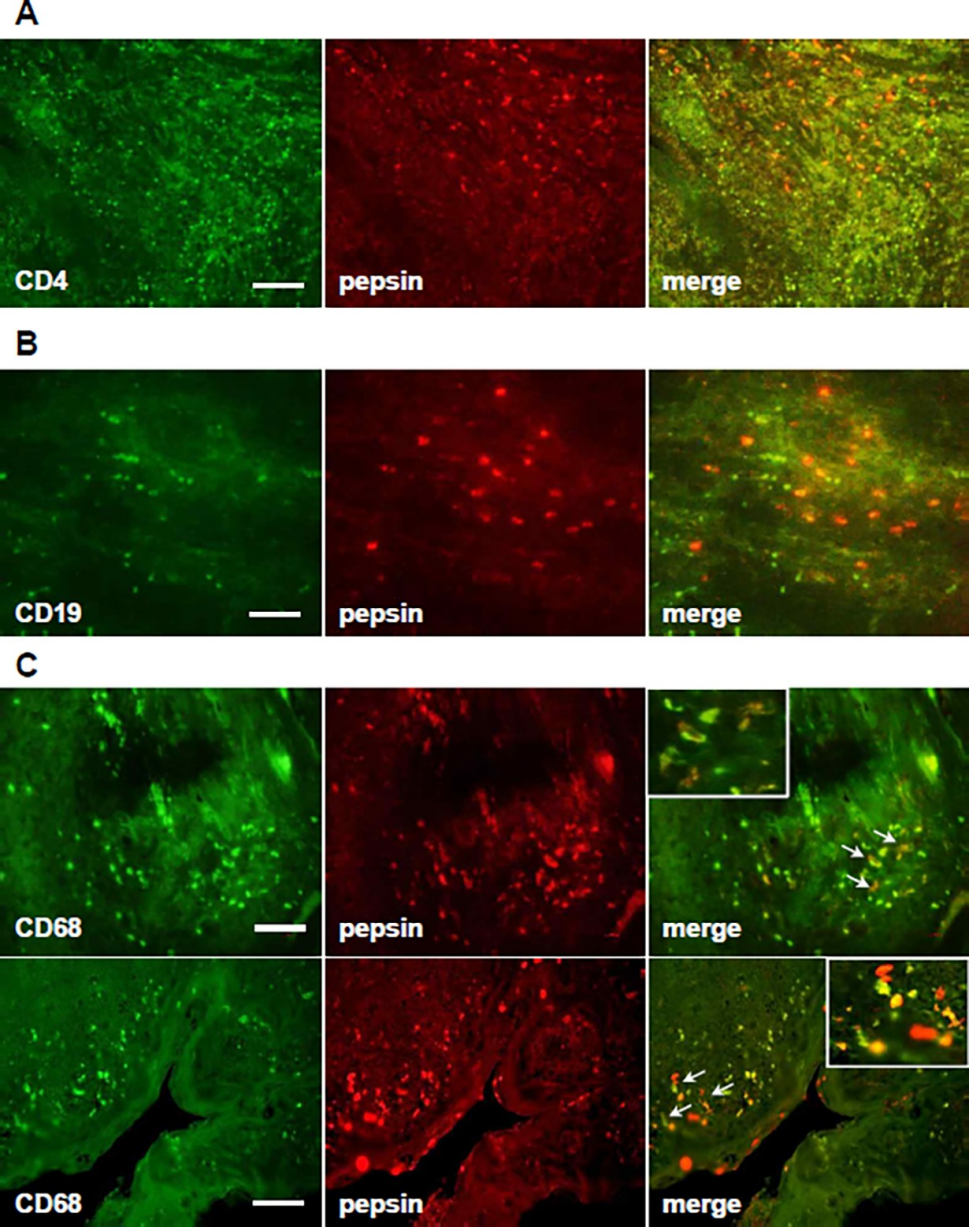
Co-localization of pepsin and lymphocytes in hypertrophic tonsil sections. No co-localization of pepsin with CD4- (A) and CD19 (B)-positive cells was detected. Co-localization of pepsin and CD68-positive cells was detected in the surrounding germinal centers (C, upper) and crypt epithelium (C, lower). Scale bar equals 50 μm.

We performed immunohistochemistry to investigate the role of pepsin in lymphocyte infiltration and proliferation in hypertrophic tonsils. Few pepsin-positive cells co-localized with CD4 cells, but pepsin-positive cells were found surrounding CD4-positive hypertrophic tonsil cells (Fig 4A). B cells, as reflected by CD19, were also same as lymphocyte (Fig 4B). Pepsin-positive cells co-localized with CD68-positive cells (Fig 4C).

## Discussion

Lymphocyte number is positively correlated with tonsil size in hypertrophic tonsils.[4, 9] Local lymphocyte stimulation also plays a role in the etiology of tonsil hypertrophy.[5–7] Lots of studies have suggested a role for bacteria in lymphocyte stimulation and tonsil hypertrophy pathogenesis.[6–8, 18] However, the number of tonsil lymphocytes can be stimulated without bacterial infection, and the specific antigens involved have not been identified.[5, 9] In our previous study, we demonstrated that extraesophageal pepsin is involved in stimulating tonsil hypertrophy, and this may be mediated by macrophages that recognize the extraesophageal pepsin as an antigen.[9]

The current study is focused on identifying the cells that respond to pepsin and understanding how to inhibit the pepsin response. Our data are the first to demonstrate that target cells in the tonsil become hypertrophic in response to pepsin. The tonsil-derived CD4^+^ proliferation by pepsin was mediated by IL-2 and IFN-γ and is inhibited by pepstatin. However, pepsin did not stimulate proliferation of CD14^+^ and CD19^+^ tonsil cells. In addition, the responsiveness to pepsin was only observed in CD4^+^ tonsil cells from pediatric patients with tonsil hypertrophy and not in CD4^+^ cells from adults with tonsillitis.

An increase in the number of lymphocytes is the most important factor in tonsil hypertrophy.[4, 5] However, the target cells, mechanism, and antigens involved in mediating lymphocyte proliferation have not been determined. Recurring lymphocyte stimuli produced by pathogenic agents and antigens can activate monocytes and macrophages in a variety of inflammatory diseases. Secreted cytokines may stimulate immune cell proliferation and enhance the immune response. We demonstrated that extraesophageal pepsin might be one of the antigens that stimulate proliferation of lymphocytes in tonsil hypertrophy.[9] A variety of other factors, including immune-response cells, cytokines, and chemokines are also involved in lymphocyte proliferation. In addition, apoptosis in lymphocytes has been studied extensively, and autoreactive T lymphocytes can be non-specifically removed to balance the lymphocyte cell population.[19, 20] The circumstances that enhance lymphocyte proliferation include inducing the factors that stimulate lymphocyte proliferation, suppressing the factors that inhibit lymphocyte proliferation, reducing the level of apoptosis that maintains lymphocyte homeostasis in normal tonsil tissue, and pathological conditions (e.g., hypertrophy).

The cytokines IL-2 and IFN-γ induce lymphocyte proliferation directly by stimulating the lymphocytes or by activating other immune cells.[21, 22] Our results demonstrate that pepsin induces IL-2 and IFN-γ secretion from hypertrophic CD4^+^ tonsil cells and that the IL-2 response to pepsin is stronger than the IFN-γ response, as shown in the relative reductions in IL-2 and IFN-γ levels in response to pepsin inhibition in Fig 3. The inhibitory effect was demonstrated by providing pepstatin both prior to and combined with pepsin (S3 Fig). We found that pepsin-mediated reflux not only damages the tonsil epithelium directly, but also stimulates tonsil macrophages and epithelial cells to produce chemokines and cytokines that activate immune cells that can damage the tonsil mucosa. Therefore, our current and previous data suggest that pepsin-mediated reflux can induce lymphocyte proliferation and autocrine activation in hypertrophic pediatric tonsil tissue.

Pepsin-mediated reflux may also suppress regulatory T cells and inhibit the inhibitory immune response in hypertrophic tonsil tissue. CD4^+^CD25^+^ tonsil T cells can suppress the proliferation of CD4^+^CD25^–^ tonsil T cells in recurring tonsillitis, producing chronic inflammation.[23] In addition, CD4^+^CD25^+^ cells isolated from blood failed to suppress the proliferation of CD4^+^CD25^–^ tonsil T cells. However, this suppression was lost in cultures containing IL-2 or when the CD4^+^CD25^+^ tonsil cells were irradiated. This suggests a critical role for CD25^+^ T cells in lymphoid tissues, which includes the tonsils, perhaps because regulatory CD4^+^CD25^+^ T cells are required to control and suppress local inflammatory reactions. Although our study does not demonstrate a relationship between pepsin and CD4^+^CD25^+^ tonsil cells, we cannot exclude the possibility that pepsin interacts with CD4^+^CD25^+^ cells in hypertrophic tonsils.

One of the functions of apoptosis is to balance the number of lymphocytes in tonsil tissue.[24–26] Apoptosis may increase in hypertrophic tonsils during cell proliferation to maintain cell homeostasis. Apoptosis of eosinophils can also decrease in patients with allergic asthma or allergic rhinitis to limit inflammation. Sensitivity to allergens is a risk factor for tonsil hypertrophy in children,[17] and some studies suggest that tonsil hypertrophy is more frequent in children with allergies.[27, 28] In addition, Onal *et al*. found an age-dependent association between apoptosis and tonsil hypertrophy.[29] We also suggest that reductions in apoptosis may result from the effects of pepsin-mediated reflux on hypertrophic pediatric tonsils. Proliferation of lymphocytes stimulated by pepsin-mediated reflux occurred more frequently in children than in adults in our study. We found no evidence that CD4^+^CD25^+^ tonsil cells or apoptosis was affected by the volume of tonsil tissue that was surgically removed. However, these potential links will be investigated more carefully in subsequent studies.

The present study demonstrates that pepsin has a potential role in the pathogenesis of reflux tonsil hypertrophy by activating lymphocytes. We also show that pepstatin can inhibit the development of reflux tonsil hypertrophy. Pepstatin is well known to be an inhibitor of aspartic proteinases such as pepsin, cathepsins D and E. Unfortunately, pepstatin is relatively insoluble and more soluble forms need to be used which might affect their inhibitory properties. Nevertheless the preventative action of pepstatin (insoluble form) upon gastric ulceration in the pylorus-ligated rat has been observed and observation also confirmed protection from pepsin induced ulceration in a rat model.[13, 15, 16] Pepsin may bind to cellular material through its active site even at a higher pH 7.0 but without showing activity and the presence of pepstatin because it is sitting on the active site cleft in effect blocks any binding hence no antigen type response is seen. However, the human study is rare. More clinical trials using pepstatin are need and more experiment with pH control to confirm the interaction of pepsin and pepstatin. It may be combined intervention with pepstatin and acid secretory inhibitors such as H2-receptor antagonists or proton pump inhibitors.

This is a first trial, using human samples in vitro, to protect tonsillar hypertrophy by pepstatin. But, more study should be done to clarify a protective role for pepstatin in reflux tonsil hypertrophy. This study suggests that inhibiting pepsin could be one of possibilities for treating tonsillar hypertrophy in children. New strategies that focus on pepsin in the refluxate might produce novel treatments for reflux tonsil hypertrophy.

## Materials and methods

### Ethics statement

We conducted this study after the approval from Gyeongsang National University Hospital Biomedical Research Institutional Review Board (# GNUHIRB-2014-02-006). All patients (or parents) gave their written informed consent after the advantages and disadvantages of the procedure prior to their inclusion in the study. We also followed the guidelines of the Declaration of Helsinki.

### Study subjects

A total of 71 patients from January 2015 to December 2016 were enrolled for this study. They visited our hospital for the surgical removal of tonsil because they suffer from sleep apnea/snoring due to tonsil enlargement or chronic inflammation at tonsils. Inclusion criteria were as follows: a diagnosis of tonsillar hypertrophy or chronic tonsillitis by physical examination or medical history. Exclusion criteria were: abscess suspected by preoperative tests, history of head and neck trauma, malignancy, radiation therapy, systemic disorder and other clinical problems. After general anesthesia, whole tonsillectomy was performed and inferior part of tonsil was selected for tissue sampling and whole blood was selected for blood sampling. None had any postoperative complication.

### Pepsin

The major human enzyme is pepsin 3 referred to in the text as pepsin and is analogous with the swine pepsin (pepsin) as used in these experiments.

### Tonsil-derived cells isolation and phenotype analysis

All tonsils were processed within 20 minutes after surgical resection. Under aseptic condition, tonsils were washed with PBS and chopped into 1–2 mm^2^ small pieces using sterilized razor blade. The chopped tissues were sequentially passed through 70 μm nylon cell strainers (BD Falcon, MA, USA) in order to obtain a single cell suspension and centrifuged at 1500 rpm for 10 min at 4°C. The isolated cells were treated with RBC lysis buffer (BioLegend, CA, USA) for 5 min at RT and were washed with PBS. For phenotyping of tonsil derived cells, 1 x 10^6^ cells were labeled with fluorescein conjugated CD4 (PE conjugated, Miltenyi Biotec, Bergisch Gladbach, Germany), CD14 (PerCP conjugated, Miltenyi Biotec) and CD19 (PE conjugated, Miltenyi Biotec) antibody for 15 min on ice and analyzed by flow cytometry (FC500, Beckman Coulter, CA, USA).

### Cell sorting and in vitro cultivation

Cells were separated using magnetic activated cell sorting (MACS, Miltenyi Biotec) system. To perform MACS separation, 5 x 10^7^ of tonsil-derived cells were resuspended in 80 μl of MACS buffer (PBS with 0.5% BSA and 2mM EDTA) and 20 ul of CD4, CD14 and CD19 microbeads (MiltenyBiotec) were added and incubated for 15 min at 4°C. The cells were washed by adding 5 ml of MACS buffer and centrifuged at 1500 rpm for 10 min. The supernatant was aspirated completely and resuspended 500 μl of MACS buffer and placed on ice. The MS column (MiltenyBiotec) was placed in the magnetic field of a MACS separator (MiltenyBiotec) and cell suspension was passed through the MS column. Unlabeled cells were collected with pass through buffer and the column was washed three times with 500 μl of MACS buffer. The column was removed from separator and immediately flushed out the magnetically labeled cells to the fresh tube by pushing the plunger into the column. Each separated cells were divided into 4 experimental groups: Basal media culture (Con); basal media with 1 μg/ml acid pepsin (1 P); basal media with 1 μg/ml acid pepsin and 0.5 μg/ml pepstatin A (1 P + 0.5 PS); basal media with 1 μg/ml acid pepsin and 1 μg/ml pepstatin A (1 P + 1 PS). In this study, all of cells were cultivated and incubated in regular pH environments (not acidic) even in treated with pepsin. But, pepsin (pepsin from porcine gastric mucosa. Sigma. #P7012) used in the experiment was prepared, as manufacturer instruction, in deionized water at 1% (10 mg/ml) and at 0.4% (4 mg/ml) in cold 10 mM hydrochloride acid. The solution at pH4.4 is stable at -20 oC and it was kept at -20 oC. Pepsin was treated on day 0 and also additionally on day 3. Pepstatin A (pepstatin A from microbial source. Sigma. #P7012) was dissolved at 1 mg/ml in 10% (v/v) acetic acid in methanol (9:1 methanol: acetic acid), heated at 60 oC, and stored at -20 oC. Working concentration (0.5–1.0 ug/ml) was followed the manufacturer instructions. All media were pre-incubated at 37°C for 30 min. Cells were maintained at 37°C in a humidified atmosphere with 5% CO_2_. To investigate the effect of pepsin on proliferation of CD4-, CD14-, and CD19-positive cells, all cells were cultured for 7 days and cell number was counted.

### Effect of IL-2 blocking on pepsin-induced CD4 proliferation

To confirm if pepsin is involved in IL-2-mediated CD4 cell proliferation, IL-2 was blocked with IL-2 blocking antibody (anti-IL-2). CD4 cells were divided into 8 experimental groups: basal media culture (CTL), basal media with 1 μg/ml acid pepsin (1 pep), basal media with 1 μg/ml pepstatin A (1 PS), basal media with 1 μg/ml acid pepsin and 1 μg/ml pepstatin A (1 pep + 1 PS) and, in addition, 20 ng/ml of anti-IL-2 (Human IL-2 antibody, MAB202, R&D systems) was treated to each group individually. All media were pre-incubated at 37°C for 30 min. All cell cultures were maintained at 37°C in a humidified atmosphere with 5% CO2. To measure the effect of anti-IL-2 on pepsin-induced CD4+ cells proliferation, each group were cultured for 7 days and cell number was counted at day 3 and day 7 using Automatic cell counter (EVETM, NanoEnTek, Seoul, Korea). To confirm if pepsin is involved in IL-2-mediated CD4 cell proliferation, IL-2 is blocked with IL-2 blocking antibody (anti-IL-2/#MAB202. R&D systems).

### Cytokine analysis

To verify cytokines on CD4-positive cells proliferation in presence or absence of pepsin, ELISA for IL-2, IFN-γ, and IL-10 was performed. After 7 days culture, culture media were collected and centrifuged at 1500 rpm for 10 min in order to spin down cells and cell debris. Fresh supernatant were collected and were kept in -80°C deep freezer. Levels of IL-2 and IFN- γ, were measured by using a specific ELISA kit (Quantikine ELISA kit, R&D Systems, MN, USA) and performed according to the manufacturer’s instructions.

### Double immunofluorescence staining

To characterize Pepsin A-positive cells, double immunofluorescence was performed on the tonsil tissues. Deparaffinization and antigen retrieval were performed. Non-specific antibody binding was blocked in PBS with 0.1% normal donkey serum (Vector Laboratories) and 0.3% Triton X-100 (Sigma) for 45 min. Sections were then incubated with anti-Pepsin A antibody (1:100; sc-99081, Santa Cruz) diluted in PBS containing 0.1% bovine serum albumin (Sigma) at 4°C overnight. After rinsing, donkey Cy3-conjugated anti-rabbit IgG secondary antibody (1:100; EMD Millipore, Billerica MA, USA) was applied for 1 hour at room temperature. For double labeling, after blocking in PBS containing 10% normal goat serum and 0.3% Triton X-100, sections were incubated with anti-CD4, CD19, and CD68 (1:100; Santa Cruz) at 4°C overnight. Alexa488-conjugated anti-mouse IgG secondary antibody (1:100; Invitrogen, Carlsbad CA, USA) was then applied for 1 hour at room temperature. Sections were mounted with anti-fading solution containing 4',6-diamidino-2-phenylindole (DAPI) (Vector Laboratories), and observed under a fluorescence microscope (Carl Zeiss Microscopy GmbH, Jena, Germany).

### Statistical analysis

All data are presented as mean ± S.E.M. Comparisons between groups were analyzed by two-tailed *t* test. Probability values (*P*) < 0.05 were considered significant.

## Supporting information

S1 Dataset Minimal data set_Rv2. (PDF)

S2 Dataset All data. (XLSX)

S1 Fig Flow cytometric analysis of peripheral blood mononuclear cells (PBMNCs) from patients with tonsil hypertrophy and tonsillitis. PBMNCs were isolated by density gradient centrifugation using a Ficoll gradient. Lymphocytes was identified using their forward and side scatter profiles and monocyte and stimulated macrophage were by staining with CD14 and CD206 antibodies, respectively. Values represent mean ± standard error of the mean (SEM). *p < 0.05 (patients <16 years, n = 40; patients >16 years, n = 20).
(PDF)

S2 Fig Purity of cell types isolated from tonsil samples using magnetic-activated cell sorting (MACS). Tonsil tissue was homogenized and washed with cold phosphate-buffered saline. Tonsil cells were incubated with beads attached to anti-CD4, CD14, and CD19 antibodies to identify T lymphocytes, monocytes, and B cells, respectively. Only minor contamination of the selected cells was observed within the negative MACS samples, and most of the selected cells were in the positive samples (>95% purity).
(PDF)

S3 Fig Effect of different pepsin and pepstatin treatments on proliferation of cells from patients. Tonsillar CD4-positive cells were isolated using MACS, treated with pepsin and pepstatin for 7 d, and counted. Pepstatin A was treated with three conditions; 1) pre-incubation with acid pepsin and pepstatin A for 30 min (pep + PS), 2) no pre-incubation (simultaneous treatment) with acid pepsin and 1 μg/ml pepstatin A (Pre x), 3) pepstatin A first for 30 min and then acid pepsin was added (1 PS → 1 pep). Fold changes were calculated relative to final values in the control group (CTL. set as “1”). Values represent mean ± SEM. *p < 0.05 (n = 46). 1 P, 1.0 μg/ml pepsin; 1 P + 1 PS, simultaneous treatment with pepsin and pepstatin; 1 PS–>1P, pre-incubation with pepstatin followed by pepsin treatment; 0.5 PS, 0.5 μg/ml pepstatin; 1 PS, 1.0 μg/ml pepstatin.
(PDF)
