# Supplementary material for: Effects of pepsin and pepstatin on reflux tonsil hypertrophy in vitro
Source: PLoS One. 2018 Nov 8;13(11):e0207090. doi: 10.1371/journal.pone.0207090 (PMC6224077; doi:10.1371/journal.pone.0207090)
Supplement: S1 Dataset — (PDF) [file pone.0207090.s001.pdf]

Characteristics of patients

	Children (N=49)	Adult (N=22)
Age – yr		
Mean	8.24	31.33
Median	8.0	30.0
Gender - no. (%)		
Male	29	9
Female	20	13
Mean tonsil size – Grade	2.5	1.0

*Grading Scale
A. Tonsil 0: Tonsils fit within tonsillar fossa
B. Tonsil 1+: Tonsils <25% of space between pillars
C. Tonsil 2+: Tonsils <50% of space between pillars
D. Tonsil 3+: Tonsils <75% of space between pillars
E. Tonsil 4+: Tonsils >75% of space between pillars

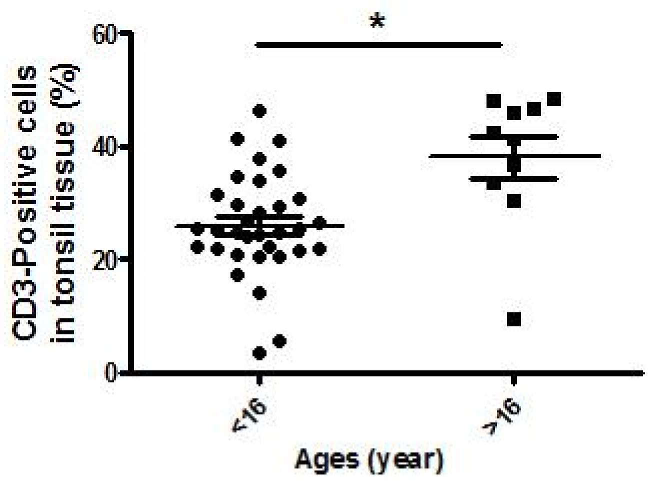

Level of CD3-positive cell in tonsil tissues with hypertrophy

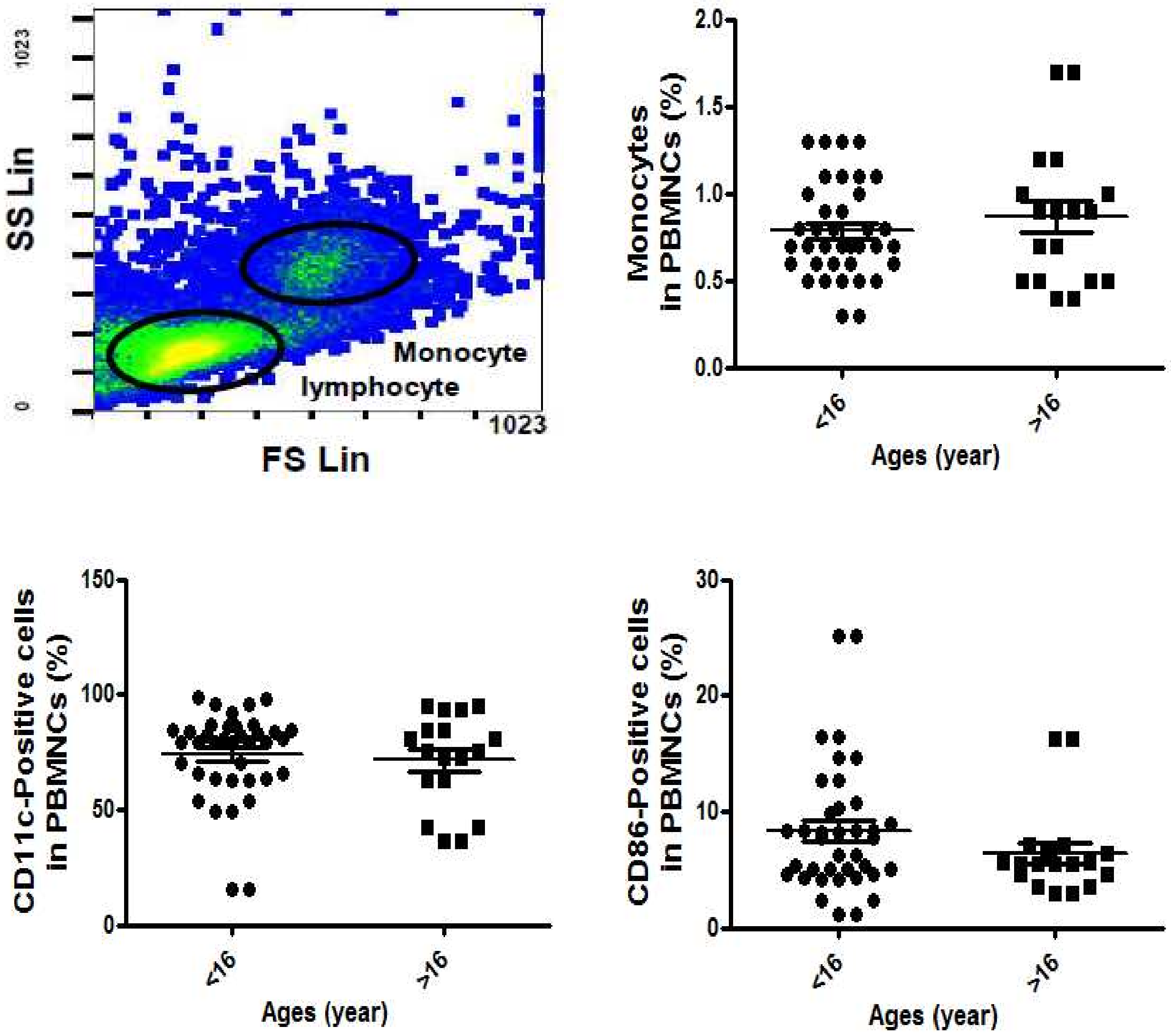

Each level from peripheral blood mononuclear cells with hypertrophy
