# Supplementary figures and images for: Effects of pepsin and pepstatin on reflux tonsil hypertrophy in vitro
Source: PLoS One. 2018 Nov 8;13(11):e0207090. doi: 10.1371/journal.pone.0207090 (PMC6224077; doi:10.1371/journal.pone.0207090)

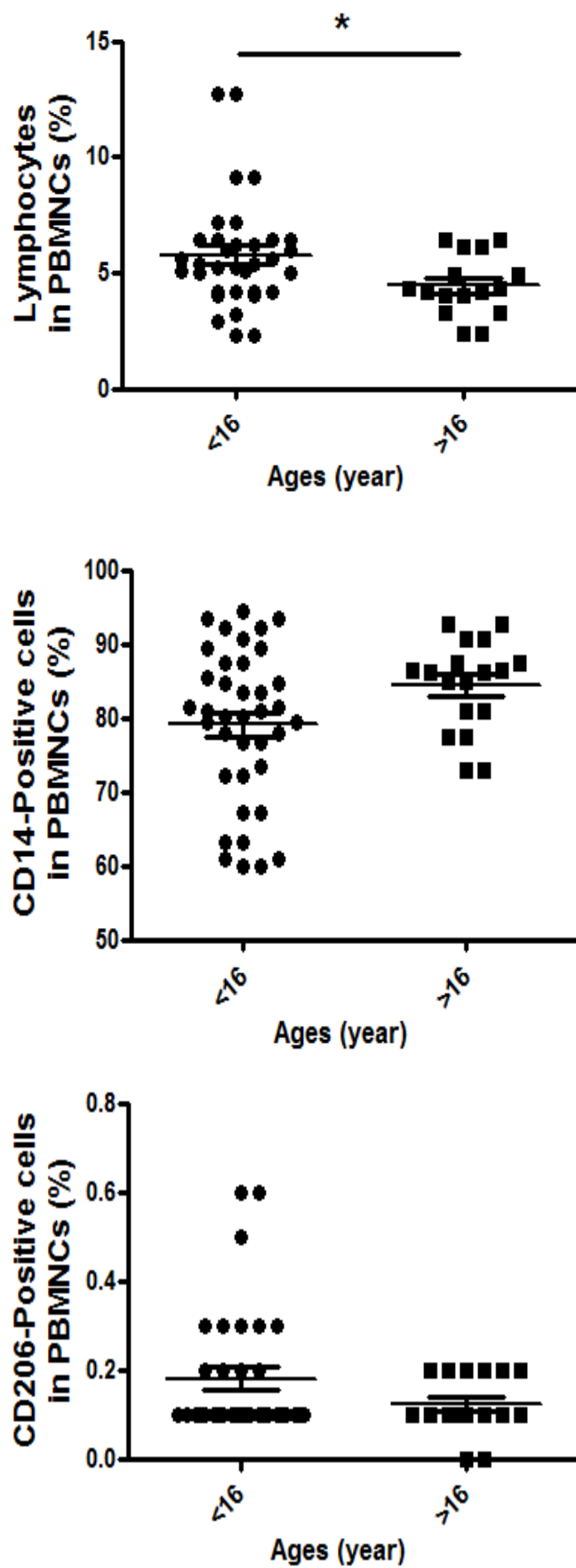

Supply Figure 1

Supplement: S1 Fig — PBMNCs were isolated by density gradient centrifugation using a Ficoll gradient. Lymphocytes was identified using their forward and side scatter profiles and monocyte and stimulated macrophage were by staining with CD14 and CD206 antibodies, respectively. Values represent mean ± standard error of the mean (SEM). *p < 0.05 (patients <16 years, n = 40; patients >16 years, n = 20). (PDF) [file pone.0207090.s003.pdf]

## Negative

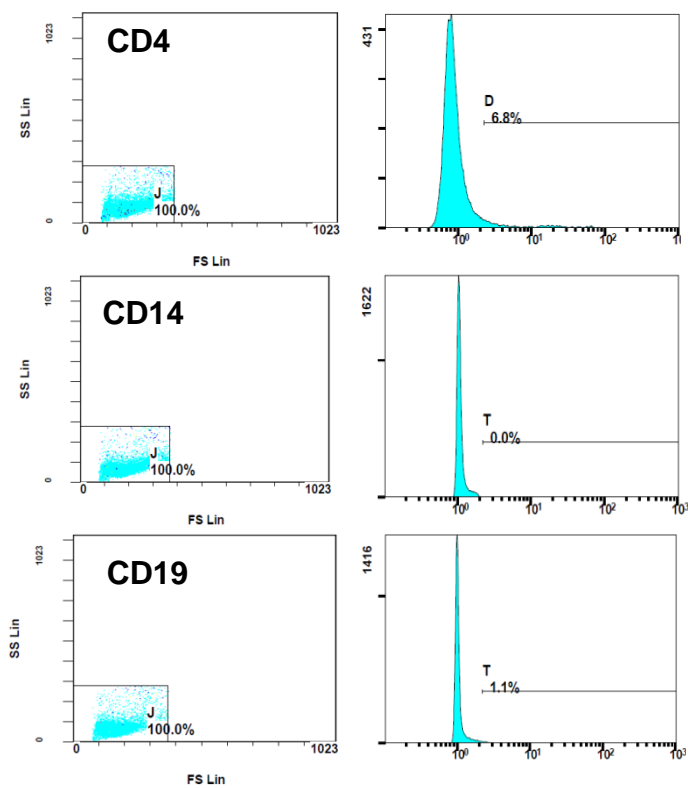

## Positive

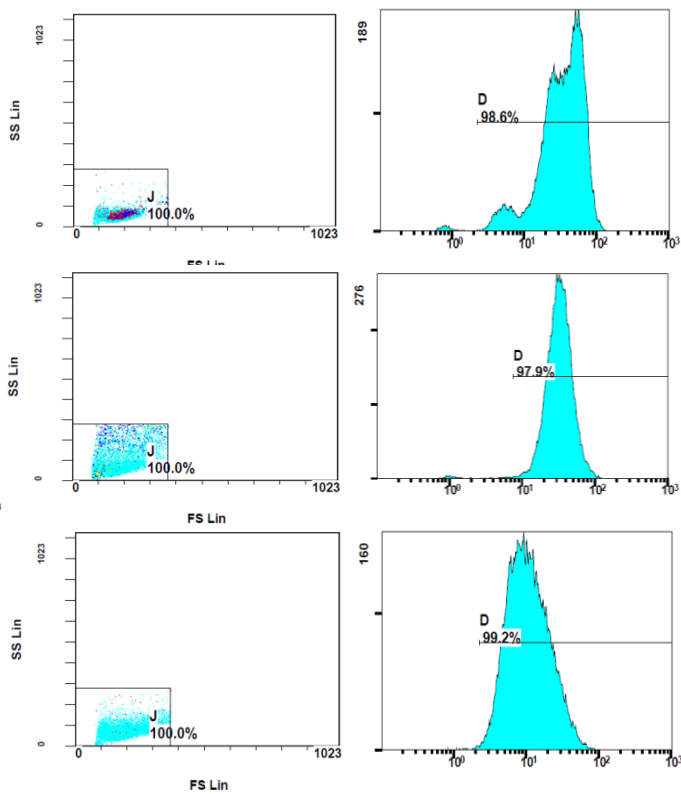

Supply Figure 2

Supplement: S2 Fig — Tonsil tissue was homogenized and washed with cold phosphate-buffered saline. Tonsil cells were incubated with beads attached to anti-CD4, CD14, and CD19 antibodies to identify T lymphocytes, monocytes, and B cells, respectively. Only minor contamination of the selected cells was observed within the negative MACS samples, and most of the selected cells were in the positive samples (>95% purity). (PDF) [file pone.0207090.s004.pdf]

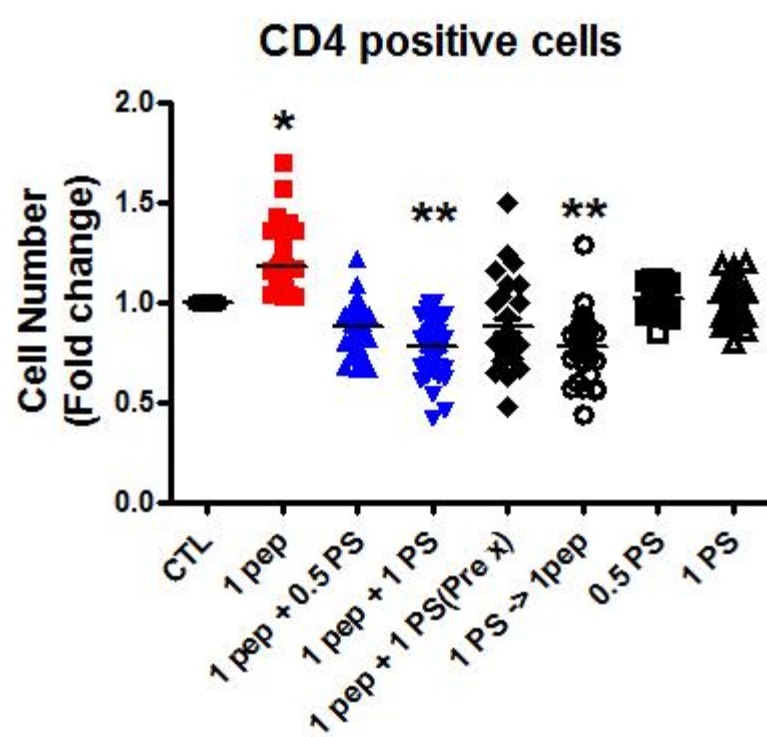

Supplement: S3 Fig — Tonsillar CD4-positive cells were isolated using MACS, treated with pepsin and pepstatin for 7 d, and counted. Pepstatin A was treated with three conditions; 1) pre-incubation with acid pepsin and pepstatin A for 30 min (pep + PS), 2) no pre-incubation (simultaneous treatment) with acid pepsin and 1 μg/ml pepstatin A (Pre x), 3) pepstatin A first for 30 min and then acid pepsin was added (1 PS → 1 pep). Fold changes were calculated relative to final values in the control group (CTL. set as “1”). Values represent mean ± SEM. *p < 0.05 (n = 46). 1 P, 1.0 μg/ml pepsin; 1 P + 1 PS, simultaneous treatment with pepsin and pepstatin; 1 PS–>1P, pre-incubation with pepstatin followed by pepsin treatment; 0.5 PS, 0.5 μg/ml pepstatin; 1 PS, 1.0 μg/ml pepstatin. (PDF) [file pone.0207090.s005.pdf]
